# Cytokine and growth factor correlation networks associated with morbidities in extremely preterm infants

**DOI:** 10.1186/s12887-024-05203-1

**Published:** 2024-11-12

**Authors:** Veronika Golubinskaya, Holger Nilsson, Halfdan Rydbeck, William Hellström, Gunnel Hellgren, Ann Hellström, Karin Sävman, Carina Mallard

**Affiliations:** 1 Department of Physiology, Institute of Neuroscience and Physiology, University of Gothenburg, Sahlgrenska Academy, 432 40530, Gothenburg, Sweden; 2 The Bioinformatics Core Facility at the University of Gothenburg, Gothenburg, Sweden; 3 Department of Pediatrics, Institute of Clinical Sciences, University of Gothenburg, Sahlgrenska Academy, Gothenburg, Sweden; 4 Institute of Biomedicine, Sahlgrenska Academy, University of Gothenburg, Gothenburg, Sweden; 5 Department of Clinical Neuroscience, Institute of Neuroscience and Physiology, University of Gothenburg, Sahlgrenska Academy, Gothenburg, Sweden; 6 Department of Neonatology, Region Västra Götaland, The Queen Silvia Children’s Hospital, Sahlgrenska University Hospital, Gothenburg, Sweden

**Keywords:** Extremely preterm infants, Retinopathy of prematurity, Bronchopulmonary dysplasia, Correlation network analysis, Cytokine interactions

## Abstract

**Background:**

Cytokines and growth factors (GF) have been implicated in the development of retinopathy of prematurity (ROP) and bronchopulmonary dysplasia (BPD). We hypothesize that even small coordinated changes in inflammatory proteins or GFs may reveal changes in underlying regulating mechanisms that do not induce obvious changes in concentration of individual proteins. We therefore applied correlation network analysis of serum factors to determine early characteristics of these conditions.

**Methods:**

Concentrations of 17 cytokines and five GFs were measured and analysed in blood samples from cord blood, on day one and during the following month in 72 extremely preterm infants. Spearman’s correlation networks distinguishing BPD and severe ROP patients from non-affected were created.

**Results:**

Most cytokine concentrations correlated positively with each other and negatively with GFs. Very few individual cytokines differed between patients with and without ROP or BPD. However, networks of differently correlated serum factors were characteristic of the diseases and changed with time. In ROP networks, EPO, G-CSF and IL-8 (cord blood), BDNF and VEGF-A (first month) were prominent. In BPD networks, IL-1β, IGF-1 and IL-17 (day one) were noted.

**Conclusions:**

Network analysis identifies protein signatures related to ROP or BPD in extremely preterm infants. The identified interactions between serum factors are not evident from the analysis of their individual levels, but may reveal underlying pathophysiological mechanisms in the development of these diseases.

**Supplementary Information:**

The online version contains supplementary material available at 10.1186/s12887-024-05203-1.

## Background

Preterm birth is a significant risk factor for neonatal morbidity [1]. Retinopathy of prematurity (ROP) and bronchopulmonary dysplasia (BPD) are common preterm birth complications.

ROP, a retinal neurovascular disease, is the main reason for blindness and visual impairment in preterm infants (more than 20,000 infants per year worldwide) [2]. The frequency of ROP needing treatment has increased with increased survival of extremely preterm infants [2]. ROP begins with reduced vascular growth and loss of existing blood vessels in the retina, followed by hypoxia-induced VEGF-related uncontrolled neovascularization with risk for retinal detachment. The influence of ROP on visual function later in life is highly variable and hard to predict. BPD is another common consequence of preterm birth found in about 40% of neonates with extremely low birth weight [3]. It is defined by the need for oxygen/respiratory support at 36 weeks of postmenstrual age, and can lead to chronic impairment of lung development and function, such as airway damage, impairment of lung vascularisation and decrease of alveolar density [3].

Low gestational age (GA) and low birth weight (BW), including intrauterine growth restriction, are known risk factors for ROP and BPD [4–6]. Intrauterine inflammation with chorioamnionitis and/or fetal inflammatory response, a significant risk factor for preterm birth, increases the risk for the development of ROP and BPD [5, 7–9]. Neonatal inflammatory processes, such as neonatal bacteremia, are risk factors for ROP [5] and BPD [10], and prolonged sterile inflammation in newborns can also contribute to the development of BPD [11]. However not all infants belonging to these risk groups develop ROP and/or BPD [12, 13]. Different risk factors likely interact, and influence of prenatal inflammation can be less pronounced if GA [7, 14, 15], mechanical ventilation in case of BPD [16], and fetal growth restriction [17] are taken into consideration. Many preterm infants develop both ROP and BPD, and both conditions likely involve response to hyperoxia and immune system dysregulation. However, it is still unclear if they appear independently or are functionally linked [18].

Biomarkers for ROP [19] and BPD [20, 21] have been sought in amniotic fluid, but since it is not readily available, blood samples are commonly used. Individual cytokines measured in blood have also been assessed. Interleukin (IL)-6 is often routinely measured in the clinic. In cord blood its levels were associated with ROP development [22, 23] and BPD [9, 24, 25], and IL-6 levels in peripheral blood taken 24 h after birth were also associated with ROP [26]. In addition, IL-8 and IL-10 in cord blood correlated with BPD in small for gestational age (SGA) preterm infants [24]. Longitudinal studies require repeated sampling over a prolonged time and larger group of patients, but provide important information on disease development and dynamic changes in blood factors. In infants with BPD, one such study showed that IL-6, IL-8 and IL-10 serum concentrations were increased during the first three days after birth [27]. In a longitudinal study of ROP development, based on the ELGAN (Extremely Low Gestational Age Newborns) cohort, increased IL-6 and TNF-α during the first month of life were associated with disease [28].

While measuring single inflammatory factors demonstrates association between their levels and disease in neonates, such analysis provides only limited information of disease mechanisms. Cytokines have complex interactions and can regulate each other’s production and release as well as activation of each other’s cellular sources [29]. Cytokines also negatively regulate growth factors (GFs), such as IGF (insulin-like growth factor)-1, in ROP infants [26]. Since cytokines can act synergistically, it is likely that concerted changes of a group of cytokines are at least as significant as large changes in individual cytokine levels. Thus, simultaneous measurement of factors is of particular interest, as shown by detailed analysis of inflammation-related networks of cytokines in amniotic fluid associated with preterm delivery and intraamniotic inflammation [30, 31]. To our knowledge, network analysis has not been applied to longitudinal postnatal data in association with preterm morbidities. In this study, we determined cytokine and GFs correlation signatures characteristic for ROP and BPD in order to identify underlying pathophysiological mechanisms for further investigation.

## Methods

### Patients

Data were retrieved from patients included in the Donna Mega study (NCT02760472) conducted at the Queen Silvia Children’s Hospital, Gothenburg, Sweden [32]. The study was approved by the Regional Ethical Board, Gothenburg (Dnr 303–11) (Clinical trial NCT02760472). Data were obtained from 90 children with GA < 28 weeks at birth admitted to neonatal intensive care. Children with major congenital malformations were not enrolled. Of the 90 children admitted, 9 died before 28 days; another three died later during the study (at days 29, 36, and 43). Children were enrolled following parental written informed consent. Cord and peripheral blood samples were taken at postnatal day 0–1, 7, 14, 28, and serum was kept frozen. Histological examination of placenta was performed according to the ELGAN protocol [33] and findings classified as histological chorioamnionitis (HCA) and/or fetal inflammatory response (FIRS) as previously described [34]. Birth weight standard deviation score (BW-SDS) was calculated according to Niklasson et al. [35].

According to international classification, retinal examinations through dilated pupils from 5–6 weeks of age were used to diagnose ROP [36]. In analyses of ROP, only infants without ROP (grade 0) or with severe ROP (ROP grade 3; 71% of them received treatment for ROP) were included. BPD was defined as the requirement for supplemental oxygen at 36 weeks of postmenstrual age. To create distinct groups with or without BPD, five infants where need for oxygen ceased close to 36 weeks (35 + 0 to 36 + 6) were excluded. The use of severe BPD in analogy with ROP was not possible as very few infants were defined as having severe BPD (five infants on CPAP, none on ventilator) at 36 weeks, and network analysis is optimal with groups of similar sizes.

### Serum concentrations of cytokines and growth factors

Cytokine concentrations were determined by a Luminex Multiplex Assay (Bio-Plex Pro Human Cytokine 17-plex Assay, Bio-Rad Laboratories, Inc., Hercules, CA) and GFs by radioimmunoassay for IGF-1 (Mediagnost GmbH, Tubingen, Germany) and ELLA multi-analyte platform for BDNF, VEGF-A, PDGF-BB, EPO (Biotechne, Minneapolis, MN). Low out-of-range values were replaced by 1/8 of the respective detection limit (Table 1S) in accordance with our previous studies [34], no high out-of-range values were identified. Concentration values were converted to log values to reduce skewness.

Where single measurements were missing and values were available from samples obtained before and after, the missing value was interpolated between the log values before and after (190 out of 5148 values interpolated). For each time point, only patients with a complete set of data for cytokines and GFs available were included in the further analysis (72 patients in total). Where more than three quarters of the values for individual cytokines were low out-of-range in any subgroup, that cytokine was excluded from further analysis; this applied to IL-4, IL-7, IL-12, IL13, and GM-CSF. For samples from days 7, 14 and 28 (“first month” time point) an average concentration was calculated as AUC/time and converted to logarithm for further analysis.

### Data analysis

Analysis of data was made in R version 4.0.2. Comparison of levels of cytokines and GFs was made by Wilcoxon’s rank sum test (R function wilcox.test) without correction for multiple comparisons.

To construct correlation networks, Spearman’s rank correlations (R function cor with method spearman) between any protein and all others were determined for each of the conditions studied (severe ROP, no ROP, BPD, no BPD) and presented as bubble plots. Correlations that differed significantly (Wilcoxon test, *p* < 0.05) between groups with and without outcomes (“difference” bubble plots) were analysed further. The protein networks were presented with their positive and negative correlations and their respective strengths marked; such a graphical map allowed a comparison between normal and affected states. In parallel, heat maps were constructed from the sum of z scores for the correlations, adding the correlation strengths for each protein into a single value showing the degree to which each protein was interacting with the others.

To correct for GA, the known main confounder, a linear regression between each protein and GA was determined for the entire group. For each patient, the deviation of the measurement from this regression line was determined for each protein, and these values were normalized to the standard deviation. Correlation networks were then constructed as above from these normalized residuals. Exposure to intrauterine infection/inflammation (HCA and FIRS) as well as intrauterine growth restriction (expressed as BW-SDS) were also analysed as possible confounders as they may influence inflammatory and GFs response in the infants [37, 38].

In all cases, *P* < 0.05 was considered significant. The details of the analysis procedure are given in Supplement.

## Results

### Clinical characteristics of patients involved in the study

Background information on the infants included in the study is presented in Tables 1 and 2; 34/72 patients were included in the BPD groups and 30/72 had severe ROP, with overlapping diagnoses in 17/72 (supplemental Fig. 1S). Infants with severe ROP or BPD had lower GA and BW compared to infants without outcomes. Deviation of birth weight (estimated as BW-SDS) was more pronounced in infants with severe ROP compared to infants without ROP, while SGA and sex did not differ. Exposure to prenatal or postnatal inflammation or preeclampsia were also equally distributed between groups.

**Table 1:** Number (*n*) of patients included in analysis of severe ROP or BPD at different time points

	**All infants**	**severe ROP**	**no ROP**	**BPD**	**no BPD**
*n, cord blood*	29	8	11	12	16
*n, day one*	70	30	15	33	35
*n, the first month*	70	30	16	32	36

*ROP* retinopathy of prematurity, *BPD* bronchopulmonary dysplasia

**Table 2:** Clinical data on all infants involved in the study and on infants divided by the presence of severe ROP or BPD

	**severe ROP**	**no ROP**	***P*-value**		**BPD**	**no BPD**	***P*-value**	
*GA, weeks*	24.9 (22.9–26.7)	27 (22.7–27.9)	*0.000*	***	25 (22.7–27.9)	25.9 (23.3–27.9)	*0.009*	**
*BW, g*	690 (415–1030)	1020 (635–1260)	*0.000*	***	693 (420–1240)	877 (415–1260)	*0.003*	**
*BW-SDS*	-0.53 (-4.82 – 0.928)	-0.16 (-2.39 – 1.65)	*0.044*	*	-1.06 (-4.21 – 1.65)	-0.49 (-4.82 – 1.56)	*0.259*	*ns*
*BW-SDS below median*	14/31	6/17	*0.555*	*ns*	19/34	18/38	*0.490*	*ns*
*SGA*	5/31	1/17	*0.402*	*ns*	8/34	4/38	*0.206*	*ns*
*Sex, boys*	19/31	13/17	*0.350*	*ns*	18/34	23/38	*0.635*	*ns*
*FIRS*	11/27	3/15	*0.306*	*ns*	11/31	15/32	*0.446*	*ns*
*FIRS or HCA*	18/26	7/15	*0.194*	*ns*	20/30	20/32	*0.795*	*ns*
*Preeclampsia*	4/31	2/14	*1.000*	*ns*	4/33	7/36	*0.518*	*ns*
*Prenatal steroids*	30/31	14/17	*0.121*	*ns*	33/34	34/38	*0.361*	*ns*
*Early-onset sepsis*	1/31	0/17	*1.000*	*ns*	1/34	1/38	*1.000*	*ns*
*IVH3-4*	5/31	2/17	*1.000*	*ns*	3/34	5/38	*0.714*	*ns*
*NEC surgically treated*	3/31	0/17	*0.543*	*ns*	3/34	0/38	*0.100*	*ns*
*Days on ventilator (median, range)*	26 (2–55)	5 (1–43)	*0.003*	**	15 (1–55)	13.5 (1–53)	*0.524*	*ns*

*GA* gestational age, *BW* birth weight, *BW-SDS* birth weight standard deviation score, *SGA* small for gestational age, *FIRS* fetal inflammatory syndrome, *HCA* histological chorioamnionitis, *IVH* intraventricular hemorrhage, *NEC* necrotizing enterocolitis, *ROP* retinopathy of prematurity, *BPD* bronchopulmonary dysplasia. Data given as median (range) or as number/total. Birth weight standard deviation score (BW-SDS) was calculated according to Niklasson et al. [35]. Statistical analysis: GA, BW, BW-SDS and days on ventilator – Wilcoxon test, the rest of comparisons – Fisher’s exact test for count data. n – number of patients per group

### ROP

#### Levels of cytokines and GFs in ROP

In severe ROP vs no ROP, IGF-1, MIP (macrophage inflammatory protein)-1β, IL-1β and IL-8 were different in cord blood, BDNF (brain-derived neurotrophic factor) and IGF-1 differed in peripheral blood on day one and IGF-1 differed in the period of first month after birth (Table 3). IGF-1 and BDNF were decreased, and MIP-1β, IL-1β and IL-8 were increased in severe ROP. After correction for GA, these differences between groups disappeared.

**Table 3:** Significantly different serum factors at the different time points

	**severe ROP vs. no ROP**					**BPD vs. no BPD**				
	*protein*			*P*-value	*P*-value	*protein*			*P*-value	*P*-value
		severe ROP	no ROP	(raw)	(corrected)		BPD	no BPD	(raw)	(corrected)
**cord blood**	*IGF-1 *	9.5 (5-38)	26 (15-50)	0.043 ↓	n.s. (0.442)	*IL-2 *	1.3 (0.19-53)	0.19 (0.19-1.86)	0.011 ↑	0.041 ↑
	*MIP-1β *	89 (32-250)	54 (11-107)	0.041 ↑	n.s. (0.351)	*IL-10*	6.1 (0.21-357)	0.21 (0.21-18)	0.008 ↑	0.027 ↑
	*IL-1β *	0.89 (0.36-14.8)	0.28 (0.06-34.4)	0.006 ↑	n.s. (0.657)					
	*IL-8 *	102 (54-1342)	47 (13-1213)	0.009 ↑	n.s. (0.968)					
**day one**	*BDNF *	1719 (276-7469)	4062 (843-10455)	0.006 ↓	n.s. (0.154)	*BDNF*	2172 (117-9654)	4075 (456-10455)	0.022 ↓	n.s. (0.077)
	*IGF-1 *	10 (2-24)	16 (4-35)	0.015 ↓	n.s. (0.682)					
**first month**	*IGF-1*	20 (11-28)	31 (10-44)	0.000 ↓	n.s. (0.100)	*IL-10*	3.3 (0.21-577)	1.9 (0.21-55)	0.022 ↑	0.035 ↑
	*EPO *	7.9 (2.7-40)	9.5 (5-22)	n.s. (0.165)	0.005 ↓					0.041 ↑

Raw data given as median (range). Arrows in *P*-value column show the direction of change. *P*-value (raw) – comparison between the groups done without correction for gestational age, *P*-value (corrected) – with correction for gestational age. *P*-value < 0.05 regarded as significant

#### ROP-related correlation networks of serum factors

**Fig. 1:**
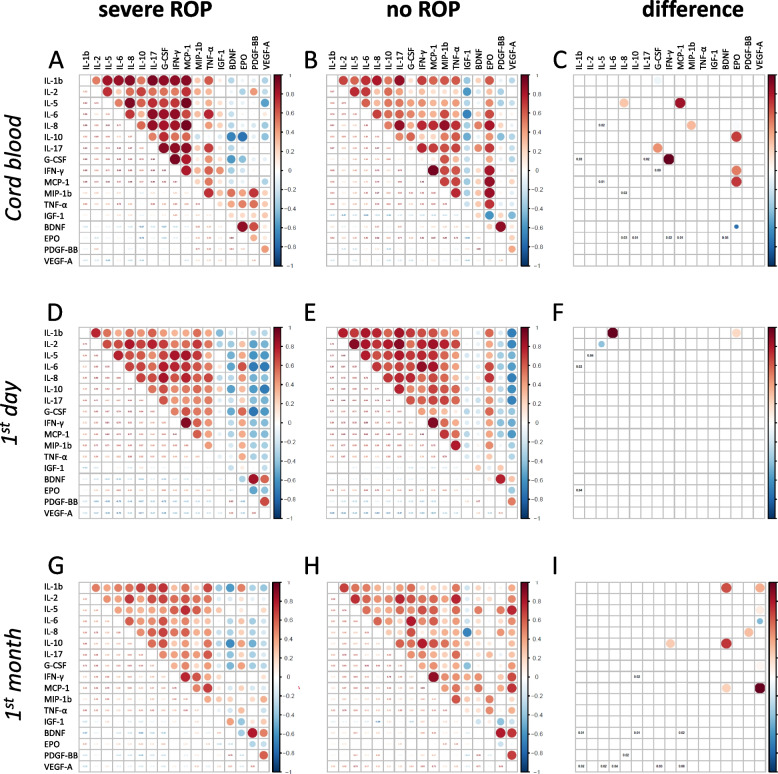
Correlations between serum factors in ROP. Bubble plots representing correlations between the levels of cytokines and GFs in cord blood (**A**—**C**), in peripheral blood within the first day (**D**—F) and the first month after birth (**G**—**I**) for patients with and without ROP. Plots in **A**, **D**, and **G** show correlations in the groups with severe ROP, and **B**, **E**, and **H** correlation in the groups without ROP. Red represents positive and blue negative correlations with deeper color for larger absolute value of correlation coefficient. The bubble size indicates significance of difference (*P* values for individual correlations in the lower part of bubble plot). Plots **C**, **F**, and **I** show the difference between severe ROP and no ROP for the corresponding period (*P* < 0.05). Correlations are based on residuals after correction for gestational age

The results of correlation analysis are presented in Fig. 1 as bubble plots for the groups with severe ROP (left panels), without ROP (middle panels) and correlations significantly different between the groups (right panels). In severe ROP, positive correlations were more often observed between cytokines, while correlations were often negative between GFs and cytokines. This pattern was evident in cord blood and peripheral blood after birth. In this context EPO (erythropoietin) behaved similarly to cytokines.

**Fig. 2:**
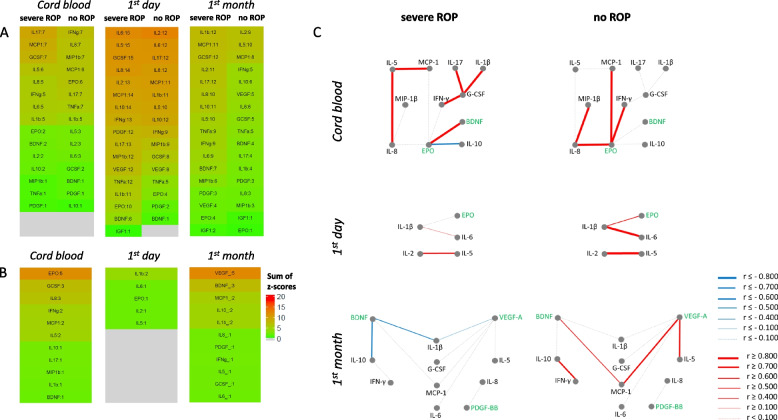
Heat maps and networks for cytokines and GFs in severe ROP. Panel A shows heat maps where serum factors have been ranked (graded by color) by the sum of the z-scores of their correlations. The number of significant correlations for the factor given after the colon. Panel B shows a similar ranking of the differences in correlations between patients with severe ROP and without ROP. Panel C shows ROP-related networks of factors with correlations different between severe ROP and no ROP. Red indicates positive, blue negative, grey non-significant correlations. Line thickness reflects correlation strength. GFs are labeled green

Correlations between factors were arranged in networks, and the weight of individual serum factors in each network was evaluated as a sum of z-scores (Fig. 2 a,b). Correlations were analysed as "entire" networks (all significant correlations within groups with and without a specific outcome) and “outcome-related” networks (only correlations significantly different between the groups with and without outcome).

Z-score sums for entire correlation networks within groups with severe ROP and without ROP are presented in Fig. 2 a; Fig. 2 b shows sum of z-scores for differences in correlation networks between these two groups. Figure 2 c shows a graphic representation of ROP-related correlation networks.

In cord blood and day one, mostly cytokines and EPO had the highest z-score. In cord blood, EPO, G-CSF and IL-8 built the most numerous and strongest connections in the ROP-related network and were descriptively identified as a “hub” factors of the network (top elements in the z-score ranking). Within the entire network in the ROP group the maximal z-score belonged to IL-17.

The ROP-related correlation network was least developed on day one, and lacked clear “hub” factors. In the latest time period cytokines still showed highest z-score in the entire correlation networks, while GFs BDNF and VEGF-A were “hub” factors in the ROP-related network.

ROP-related networks before and after correction for GA were comparable in complexity (Fig. 2, Suppl. Figure 2S). The ROP-related network during the first month of life was the least affected by GA correction.

#### Analysis of possible confounders in ROP-related networks

**Fig. 3:**
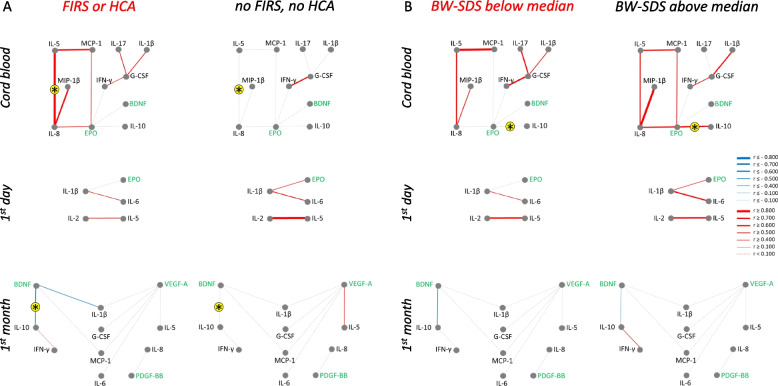
ROP-related networks for serum factors in patients with and without various diagnoses. Networks identified for severe ROP (Fig. 2) were analyzed in patients grouped by exposure to intrauterine inflammation (histological chorioamnionitis (HCA) and/or fetal inflammation (FIRS) based on placenta examinations, panel A) or fetal growth (birth weight standard deviation score (BW-SDS) panel B) at different time points. Same marking of networks as in Fig. 2 C. Asterisks on yellow background show significant differences between patients with and without the respective condition

We analyzed whether possible confounders, such as prenatal inflammatory exposure (groups with or without HCA and/or FIRS) or intrauterine growth restriction (BW-SDS below or above median) influenced the ROP-related correlation networks (Fig. 3). Very few correlations that were associated with ROP were also significantly different between these groups (marked with asterisks in the figure). Correlations between IL-8 and IL-5 in cord blood and between IL-10 and BDNF during the first month were stronger in the group with FIRS or HCA. Correlations between EPO and IL-10 in cord blood were stronger in the group with BW-SDS above median.

### BPD

#### Levels of cytokines and GFs in BPD

In BPD vs no BPD, IL-2 and IL-10 were higher in cord blood, BDNF was lower on day one and IL-10 was higher in the first month after birth (Table 3). After correction for GA, the differences in BDNF level became non-significant (although near the limit), but the increase in IL-2 and IL-10 remained.

#### BPD-related correlation networks of serum factors

**Fig. 4:**
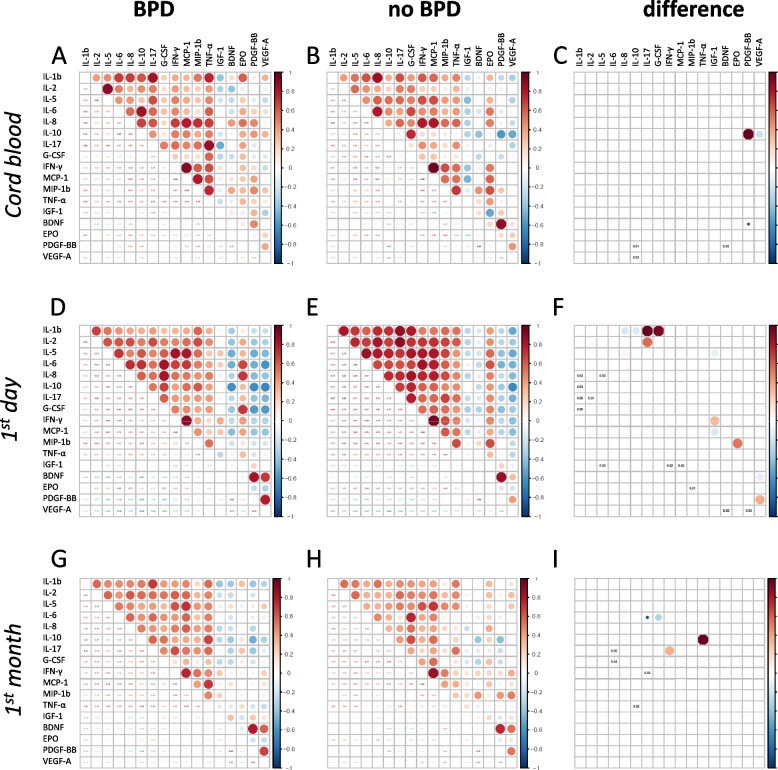
Correlations between serum factors in BPD. Bubble plots representing correlations between levels of serum factors (cytokines, GFs) in cord blood (**A**—**C**), in peripheral blood within the first day (**D**—**F**), and during the first month after birth (**G**—**I**) for patients with or without BPD. Factors significantly different between groups are presented in (**C**, **F**) and (**I**) plots (*P* < 0.05). Values are corrected for gestational age. See Fig. 1 for a detailed description

Figure 4 presents results of correlation analysis as bubble plots for the groups with (left panels), without (middle panels) BPD, and correlations significantly different between the groups (right panels). Positive correlations were observed more frequently between cytokines, and negative correlations between GFs and cytokines. EPO behaved similarly to cytokines.

**Fig. 5:**
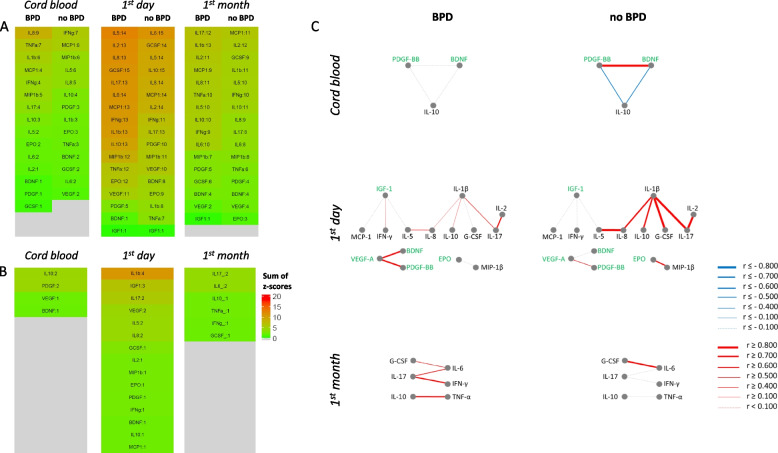
Heat maps and networks for cytokines and GFs in BPD. Panel (**A**) shows heat maps in patients with or without BPD; panel (**B**) shows a similar ranking of the differences in correlations between these patients. Panel (**C**) shows BPD-related networks—same presentation as in Fig. 2

The weight of individual serum factors calculated as a sum of z-scores in correlation networks and graphic representation of BPD-related networks are shown in Fig. 5. Z-score sums for entire correlation networks within groups with or without BPD are presented in Fig. 5a, z-score sums for differences in correlation networks between these two groups in Fig. 5b. Figure 5c shows a graphic representation of BPD-related correlation networks. Groups with and without BPD differed on day one and during first month, but in opposite direction. On day one cytokines showed stronger correlations within the no-BPD group, but during the first month correlations were more pronounced in the BPD group. In the total network heatmaps (Fig. 5a), only cytokines were found as highly ranked “hub” factors. BPD-related network (Fig. 5b, c) in cord blood was the least developed and did not have clear “hub” factors compared to the other time points, and correlations were stronger in the no-BPD group. In blood from day one, IL-1β, IGF-1 and IL-17 were “hub” factors, and in the first month there were no clear “hub” factors in BPD-related networks.

BPD-related networks were comparable in complexity before and after correction for GA (Fig. 5, Suppl. Figure 2S).

#### Analysis of possible confounders in BPD-related networks

**Fig. 6:**
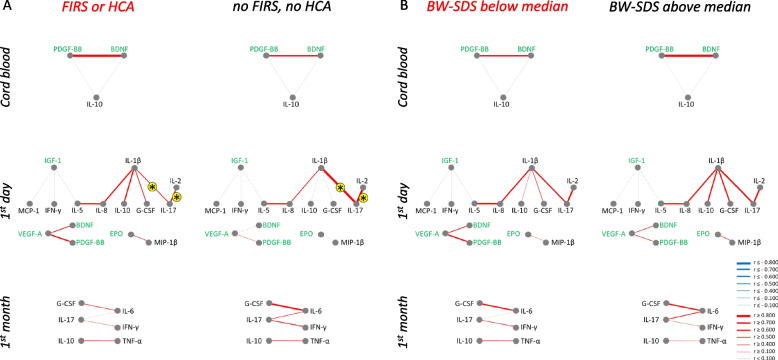
BPD-related networks for serum factors in patients with and without various diagnoses. Networks identified for BPD (Fig. 5) were analyzed in patients grouped by presence of placental and/or fetal inflammation (FIRS or HCA, panel **A**) or bodyweight deviation (BW-SDS, panel **B**) at different time points. Same marking of networks as in Fig. 2C. Asterisks on yellow background show significant differences between patients with and without the respective condition

The relationship between the factors involved in BPD-related networks in infants with or without prenatal inflammatory exposure (with or without HCA and/or FIRS) and intrauterine growth restriction (BW-SDS below or above median) is presented in Fig. 6, correlations significantly different between these groups are marked with asterisks. In general, BPD-related correlation signatures were affected very little by these factors. Only the connections of IL-17 with IL-1β and IL-2 on day one were affected by perinatal inflammation.

## Discussion

In the present study we describe characteristic signatures for cytokines and GFs in relation to ROP and BPD in cord blood, peripheral blood at first day and during first month of life. Signatures were based on correlation network analysis and demonstrate distinct characteristics for these two important morbidities. The rationale for the present study was that even small coordinated changes in inflammatory proteins or GFs may reveal changes in underlying regulating mechanisms that do not induce obvious changes in concentration of individual proteins. Network analysis, while often used for big data, may have an advantage also in case of only a few, but simultaneously measured parameters, and it can reveal characteristic patterns of possible mechanistic importance for further studies.

Very few serum factor concentrations differed after correction for GA, and there are currently no reliable blood markers for the diseases. However, the outcome-specific networks showed specific morbidity-related patterns that changed over time after birth. Concentrations of cytokines showed positive correlations with each other and negative correlations with GFs; GFs were less involved in outcome-related networks than cytokines. In ROP-related networks EPO, G-CSF, IL-8, BDNF and VEGF-A formed a number of strong connections at different time points, while in BPD IL-1β, IGF-1 and IL-17 emerged as network hubs.

In addition to GA, we analysed fetal inflammation and fetal growth restriction, since intrauterine exposure to infection/inflammation as well as chronic fetal hypoxia resulting in fetal growth restriction may influence fetal and neonatal protein response [37, 38]. The analysis showed a limited influence of these confounders, with networks mainly determined by the disease.

### Correlation network analysis

Network analysis considers factors as a continuum where individual parameters affect each other by up- and downstream regulatory connections. Even small, but simultaneous changes in concentrations of a few factors in the same functional network may result in a significant change of function. Currently there are very few studies using a network-based approach to analyse cytokines or GFs in the field of neonatal research. However, network-based analysis of blood inflammatory proteins resulting in “signatures” and “hub” factors may be of particular importance in understanding inflammation-related pathophysiology in preterm infants. Using a similar approach, network analysis of amniotic fluid in patients with spontaneous preterm labour revealed networks related to intra-amniotic inflammation [30, 31].

### Cytokine correlations

We found positive correlations between cytokines, whether pro- or anti-inflammatory, in all patient groups, likely indicating a general inflammatory response. Increased correlations between pro-inflammatory cord blood cytokines have been shown in a mixed term-preterm group of neonates, while anti-inflammatory IL-5 and IL-13, and pro-inflammatory IL-17 did not correlate with other cytokines [39]. However, in the present study IL-5 and IL-17 connected to other cytokines in both ROP and BPD networks.

### Networks associated with ROP and BPD

Networks were clearly different between ROP and BPD groups and were also changing over time: the most developed BPD network was observed on day one after birth, while for ROP most correlations were observed in cord blood and during first month. Several proteins appeared repeatedly in consecutive networks, but mostly with new connections. Network differences between cord blood and blood from day one likely reflect the difference between the intrauterine environment and the infant’s response to postnatal challenges, while later networks may be influenced by developmental changes, morbidities and clinical interventions. In general, the number of factors involved in the networks was comparable between ROP and BPD, but it happened at different time points.

Cytokines are important inflammatory mediators, and both ROP and BPD are associated with exposure to intra-uterine inflammation [5, 7–9]. However, we observed very little association with placenta findings of infection/inflammation, which suggests that other risk factors influence the inflammatory response and its association to morbidities.

There were pronounced changes in heatmaps of the entire networks (Figs. 2a, and 5a) between cord blood and day one, the z-scores for most of the factors were increased on day one. This might reflect inflammatory response at birth and a modulating influence of the intrauterine environment on protein responses. A number of factors repeatedly high in the heatmaps may be of specific importance. Importantly, for many of the hub factors their concentrations alone were not significantly different between the groups, which is also consistent with the network analysis reported by Romero et al. [30].

“Hub” cytokines in the disease-associated networks at early time points of life include IL-1β (BPD on day one) and IL-8 and G-CSF (ROP, cord blood). All these cytokines are known to be proinflammatory, but they are not very much studied in relation to ROP or BPD. IL-1β levels increase in blood serum [40] and bronchoalveolar fluid [40–42] in infants developing BPD, and it has been suggested to be involved in pulmonary inflammatory response and in BPD development [41, 43]. Increased vitreous and blood levels of IL-8 have been observed in association with ROP [28, 44–46], but did not show sufficient power as predictor for ROP [44]. Data about G-CSF involvement in ROP is controversial: G-CSF vitreous levels were increased in ROP patients and are associated with ROP-related inflammation [47, 48], and G-CSF deficiency had a partial protective effect in animal model of ROP [49], but at the same time G-CSF can be given to premature infants as a clinical treatment, and in such infants there was a trend (though insignificant) for reduced need in laser treatment for ROP [50]. The results of our study suggest that these proinflammatory factors can be considered for more detailed study as potential players in preterm birth-related morbidities.

### GF correlations

In the present study, not only cytokines, but also growth factors were present in the networks. Interestingly, in the noROP group during the first month, the critical neovascularisation factor VEGF-A formed positive correlations with IL-5 and with chemokine MCP-1. While the importance of cytokines for ROP has been studied previously [19, 22, 23, 26, 28], MCP-1 may be a new player in ROP development. MCP-1 is very little studied in relation to ROP. In a model of neonatal hyperglycemia-induced retinopathy, MCP-1 increase was associated with recruitment of inflammatory macrophages and microglia to the eye and reduction of retinal vascular area [51]. In our data, a positive correlation between MCP-1 and VEGF-A is observed only in infants without ROP, suggesting that in the healthy eye VEGF elevation during the neovascularisation stage of ROP induces MCP-1-dependent removal of badly formed blood vessels, a mechanism lost in infants developing severe ROP. The fact that such correlations are present in our data only at later time points may indicate that they are involved in ongoing neovascularisation. Further support of this hypothesis is that MCP-1 is correlated not only with VEGF, but also with BDNF. It has been demonstrated that BDNF and VEGF levels show similar dynamics in preterm infants with ROP [52], and that BDNF might be involved in the development of diabetic retinopathy [53]. Thus, the relation between VEGF, MCP-1 and BDNF should be studied in experimental models for ROP as well as in the clinical setting.

Unlike other GFs, EPO was generally positively correlated with cytokines (Figs. 1 and 4), especially at early time points. EPO is often considered an anti-inflammatory cytokine [54], which can explain its positive correlations with other cytokines in our study. EPO levels are low in blood plasma of preterm infants compared to term infants [55], but can increase in amniotic fluid and fetal plasma in situations with fetal hypoxia [56]. In our outcome-related networks, it could be found in ROP in cord blood as a clear “hub” factor, and on day one in both ROP and BPD, although not with high z-scores (Figs. 2 and 5). In ROP-related networks in cord blood EPO builds positive connections in the no-ROP group with IL-8, MCP-1 and IFN-γ, while in the severe ROP group EPO shows positive correlation with BDNF and negative correlation with IL-10. On the day one EPO positively correlates with IL-1β in the no-ROP group. EPO, IL-8, IL-1β and IL-10 are related to cell hypoxia [57–59]. The change in correlations between hypoxia-related serum factors in our group of infants with severe ROP might signal dysregulation of the response to hypoxia. It is also possible that fetal hypoxia was more common in our ROP group, as SGA infants tended to be overrepresented there (Table 2). This would be in agreement with our observation of significantly different connections between EPO and IL-10 in infants with BW-SDS above or below median (Fig. 3), based on the assumption that lower BW-SDS is associated with placental insufficiency and hypoxia.

### Concentration levels of cytokines and GFs in ROP and BPD

In ROP, several GF concentrations differed from control, but all these differences disappeared after correction for GA (Table 3). This is in agreement with reported dependence of GF concentrations on GA [52]. In particular, IGF-1 levels correlate with GA [60, 61], as in the present study. IGF-1 and its regulators have been well studied in ROP. Reduced early postnatal serum levels of IGF-1 predicted later ROP development [62], eye development, and visual impairment [63]. Intravenous infusion of IGF-1 alone or combined with IGF-binding protein 3 was suggested as a treatment for ROP [64, 65].

In BPD, we noted elevated levels of IL-10 in cord blood and in peripheral blood during the first month after birth, but not on day one, and these elevations remained significant after correction for GA. There is quite consistent data on IL-10 concerning lung dysfunction in preterm infants. IL-10 blood levels were increased during the first three days after birth starting from cord blood and correlated with the duration of mechanical ventilation [27]. Elevated blood levels of IL-10 were associated with pulmonary hypertension in newborns [66]. Even in the mixed group of term and preterm patients, IL-10 in cord blood was predictive for respiratory distress syndrome [39]. IL-10 was also elevated in tracheal aspirates of BPD-patients during the first month after birth [67]. In the present study, IL-10 appeared in all BPD-related correlation networks: in cord blood it interacted only with GFs, and at later time points it showed connections with strong pro-inflammatory cytokines IL-1β and TNF-α. This suggests that IL-10 has different roles in BPD over time.

To investigate the effect of the combination of diseases, the group of patients with both BPD and severe ROP was compared to patients with only severe ROP at the two later time points (Supplemental Fig. 5S). The results of this comparison suggest that the presence of multiple diseases in the group of sick patients causes changes in ROP-related networks, in particular they become more pronounced and complex at day one after birth with more involvement of GFs. Unfortunately, our study group was too limited in size to permit the study of the influence of ROP in the presence of BPD, or to analyse cord blood.

Our study has some limitations. The number of patients is moderate, and the observed signatures therefore need validation in a larger cohort in future studies. In a larger cohort, also paired analysis would be possible, making conclusions regarding factors involved in pathophysiology of critical clinical conditions stronger. This study was aimed at finding early signs of ROP or BPD, but since the definite diagnosis for either disease is made at a later time point, later postnatal events (e.g. septic infections) after the currently studied period may also affect the disease development. The number of patients with severe ROP in the present study was almost twice that of those without ROP, which may influence the significance of the differences. Most cytokines are at low levels under unstimulated conditions, causing a number of measurements being below the detection limit. One should also keep in mind that in our study the proteins were measured in blood and not within the affected tissues. In addition, patients receive treatment and blood/plasma transfusions throughout the study, which may interfere with endogenous levels of cytokines and GFs.

## Conclusions

In summary, we demonstrate that analysis of correlations between cytokines and GFs can identify signatures related to ROP and BPD that are not revealed by individual protein measurements. The results of this study suggest meaningful interactions between serum factors that are not evident from the analysis of their individual levels. Although this is a small study, requiring validation in future work, this approach may reveal information on underlying pathophysiological mechanisms of the respective disease.

## Supplementary Information

Supplementary Material 1.

## Data Availability

The datasets generated during and/or analyzed during the current study are not publically available due to the requirements of the ethical permit.

## Abbreviations

BPD: Bronchopulmonary dysplasia
ROP: Retinopathy of prematurity
GF: Growth factor
GA: Gestational age
BW: Birth weight
SGA: Small for gestational age
BW-SDS: BBirth weight standard deviation score
HCA: Histological chorioamnionitis
FIRS: Fetal inflammatory response
IGF: Insulin-like growth factor
MIP: Macrophage inflammatory protein
PDGF: Platelet-derived growth factor
EPO: Erythropoietin
IFN: Interferon
VEGF: Vascular endothelial growth factor
IL: Interleukin
TNF: Tumor necrosis factor
