# Supplementary material for: Cytokine and growth factor correlation networks associated with morbidities in extremely preterm infants
Source: BMC Pediatr. 2024 Nov 12;24:723. doi: 10.1186/s12887-024-05203-1 (PMC11555815; doi:10.1186/s12887-024-05203-1)
Supplement: Supplementary file 1 — Supplementary Material 1. [file 12887_2024_5203_MOESM1_ESM.docx]

| 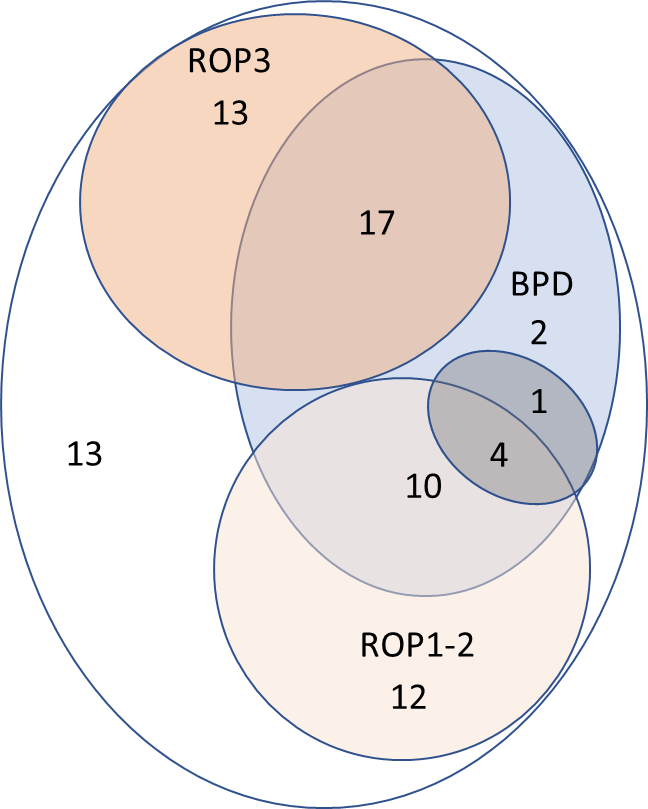 | **Supplement Fig.1S** Number of patients diagnosed with BPD, ROP of different grades, and without these diagnoses. A substantial overlap between BPD and ROP was observed.  Grey area corresponds to the infants where need for oxygen ceased close to 36 weeks (35+0 to 36+6). |
| --- | --- |

| 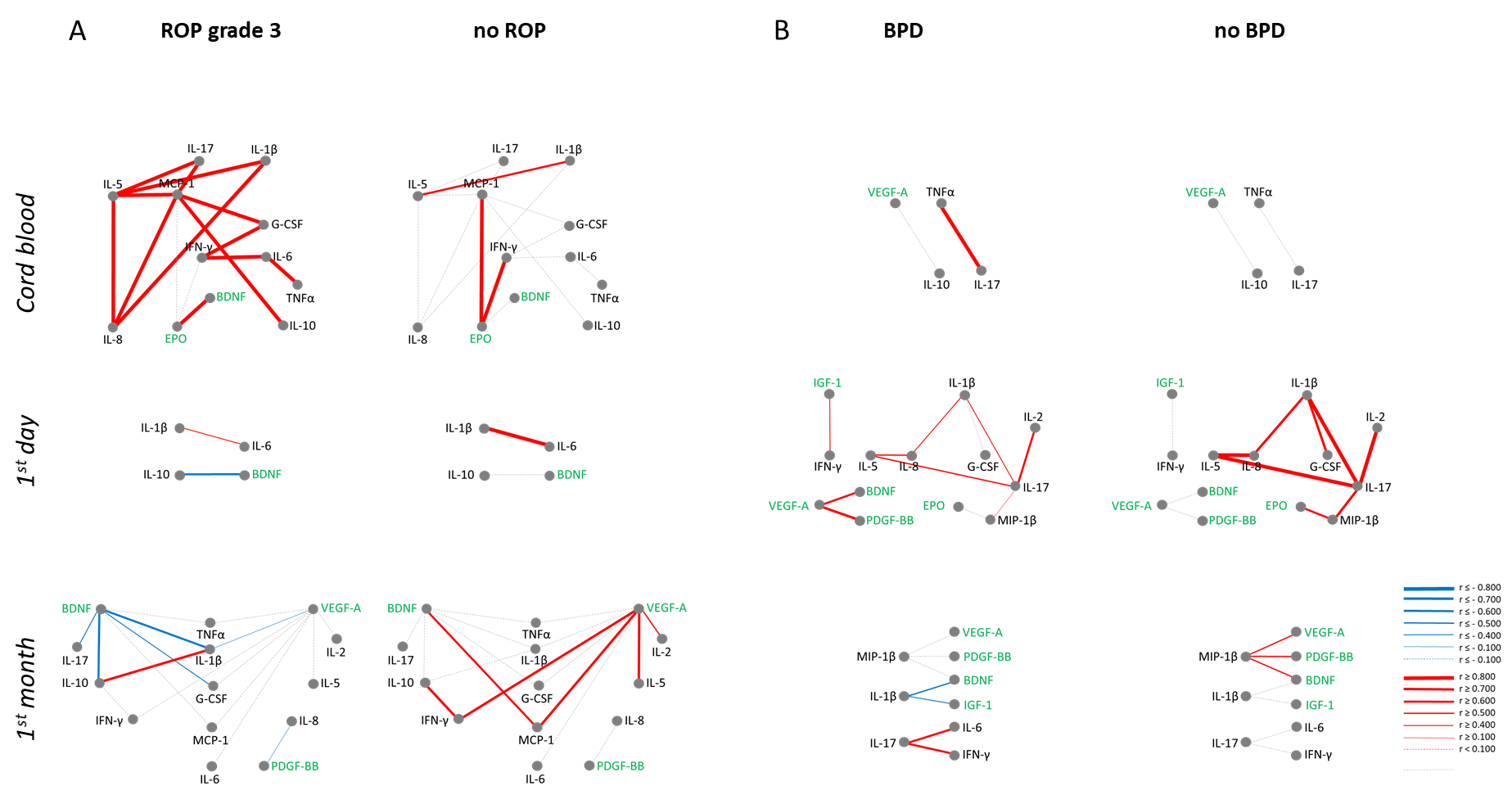 |
| --- |
| **Supplement Fig.2S** ROP- and BPD-related networks of plasma factors whose correlations were significantly different between groups with and without outcomes. Here analysis has been done without corrections for gestational age. Red shows positive correlations, blue negative; line thickness shows strength of correlation, grey lines show non-significant correlations. Growth factors are labeled green |

| 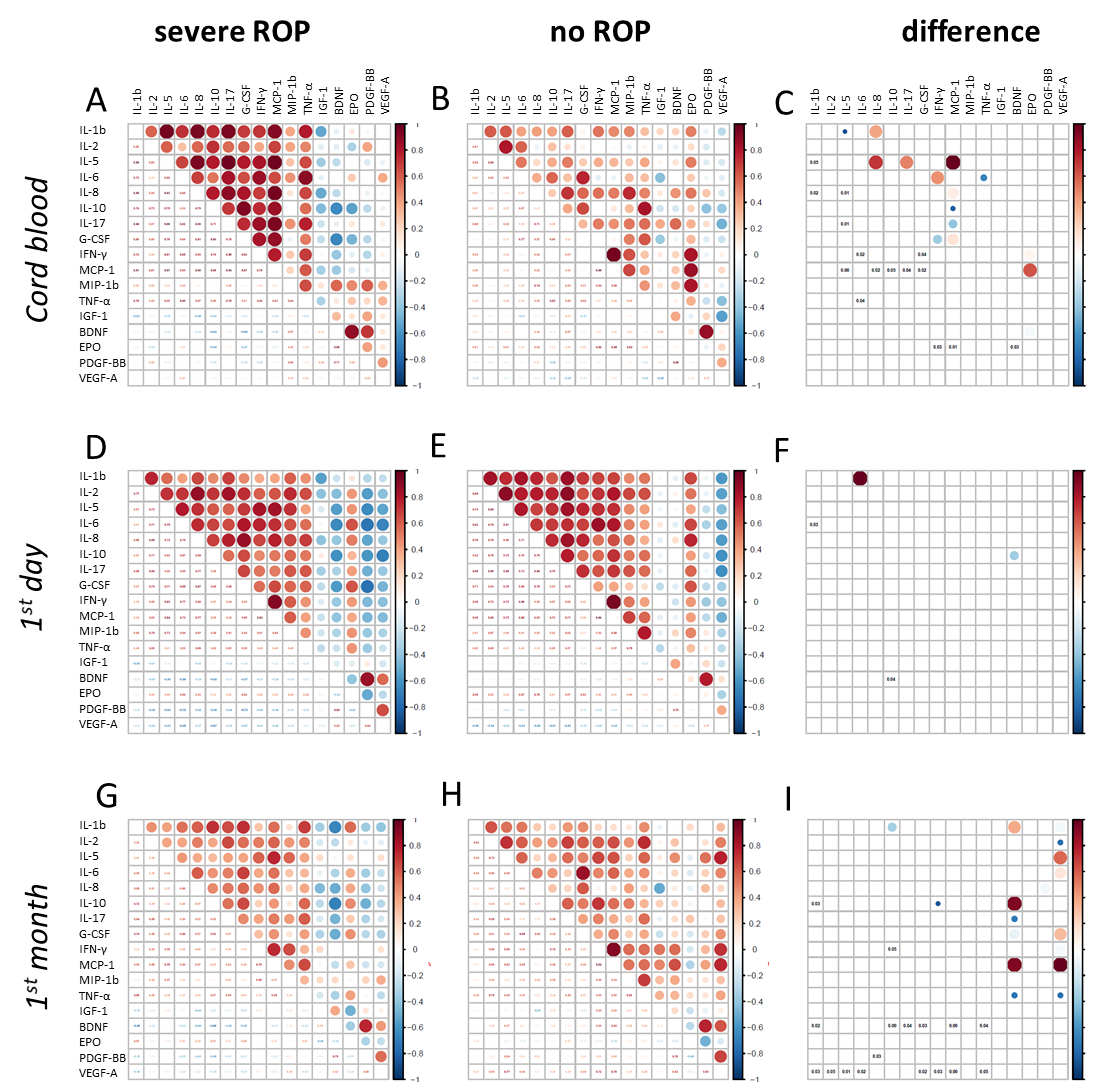 | **Supplement Fig.3S** Bubble plots representing correlations between the levels of analysed serum factors (cytokines/chemokines, growth factors) in cord blood (A - C), in peripheral blood within first day after birth (D - F) and in peripheral blood during first month starting from day 3 after birth (G - I) for patients with severe ROP and without ROP .  Plots in A,D and G show correlations in the groups with severe ROP, in B,E and H the corrresponding conrtrol group (with no ROP); color of the bubbles in these plots describes the correlation coefficient: red for positive and blue for negative correlations, the deeper the color the stronger the correlation (larger absolute value of correlation coefficient). The size of the bubbles and the values given in the lower part of each bubble plot show P values for the individual correlations within the analyzed group. Plots C,F and I show the difffernce between severe ROP and no ROP for the corresponding time period. Only significant correlations (P<0.05) are shown in red colors.  Values are NOT corrected for gestational age. |
| --- | --- |

| 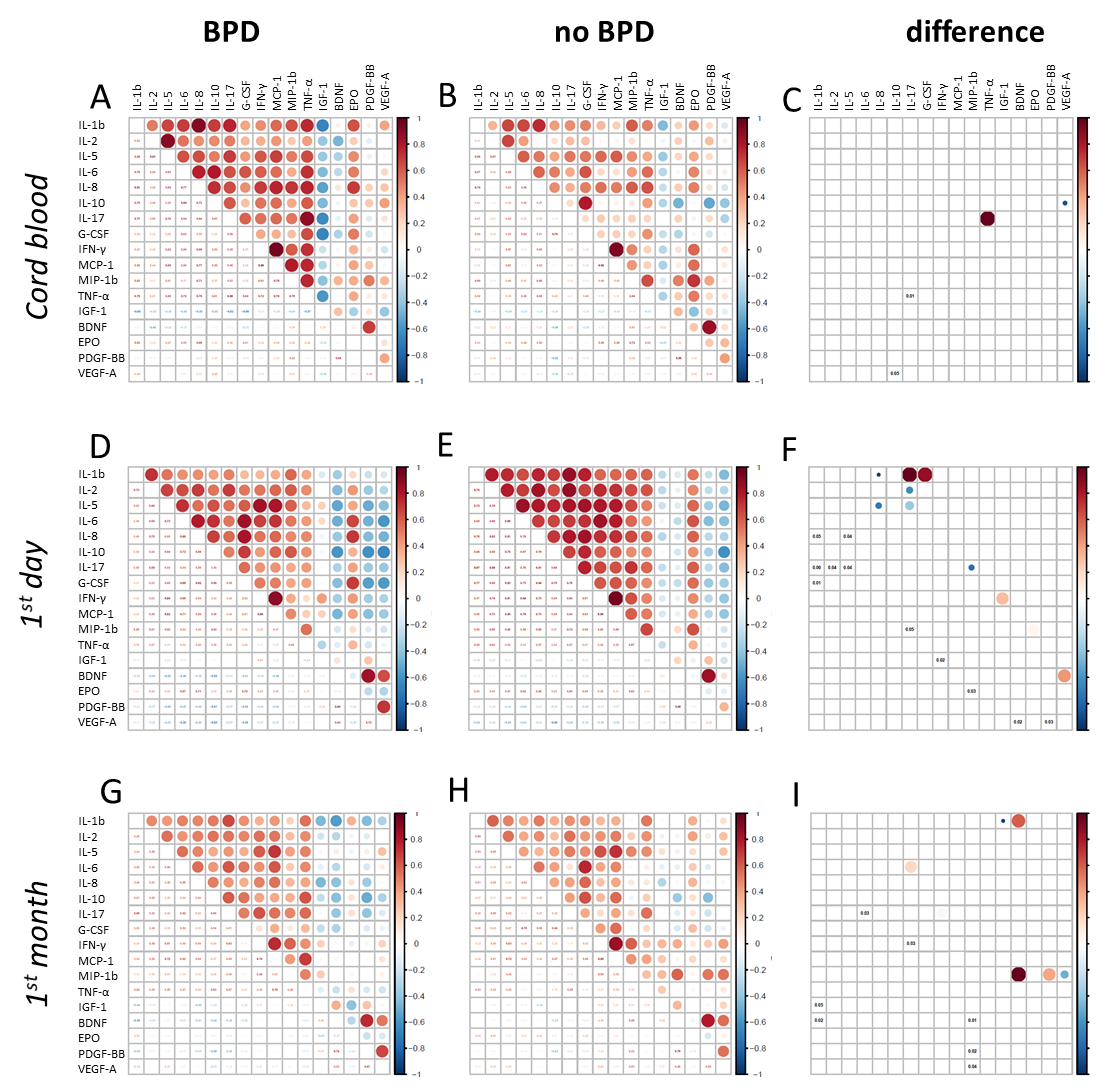 | **Supplement Fig.4S** Bubble plots representing correlations between the levels of analysed serum factors (cytokines/chemokines, growth factors) in cord blood (A - C), in peripheral blood within first day after birth (D - F) and in peripheral blood during first month starting from day 3 after birth (G - I) for patients with and without BPD .  Factors significantly different between the groups are presented in C, F, I plots (P<0.05).  See more details in Fig.1 description.  Values are NOT corrected for gestational age. |
| --- | --- |

| 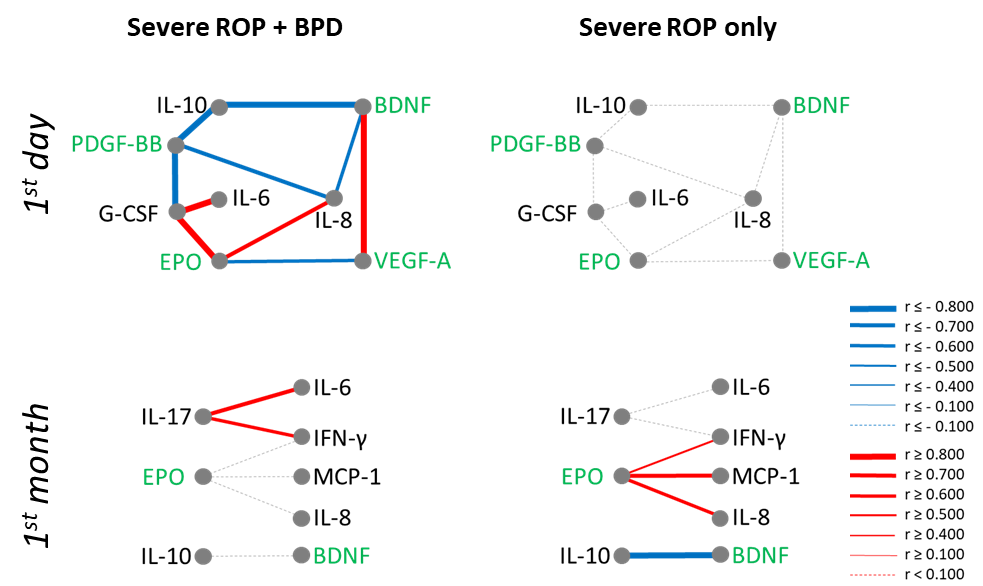 | **Supplemental Fig 5S** Networks of plasma factors whose correlations were significantly different between patients with only severe ROP and patients with simultaneous BPD and severe ROP. Analysis was done with correction for gestational age. Red shows positive correlations, blue negative; line thickness shows strength of correlation, grey lines show non-significant correlations. Growth factors are labeled green. Networks for cord blood could not be constructed, since there were too few patients in that group. |
| --- | --- |

**Supplement Table 1S**. The table shows the percentage of measurements that were below the limit of detection for the measured proteins.

|  | **Cord blood** | **Day one** | **First month** |
| --- | --- | --- | --- |
| **IL-1β** | 3 | 38 | 45 |
| **IL-2** | 52 | 35 | 51 |
| **IL-5** | 18 | 26 | 37 |
| **IL-6** | 0 | 4 | 9 |
| **IL-8** | 0 | 1 | 1 |
| **IL-10** | 39 | 19 | 33 |
| **IL-17** | 55 | 49 | 45 |
| **G-CSF** | 45 | 29 | 71 |
| **IFN-γ** | 0 | 1 | 0 |
| **MCP-1** | 0 | 0 | 0 |
| **MIP-1β** | 0 | 0 | 0 |
| **TNF-α** | 0 | 0 | 0 |
| **IGF-1** | 0 | 0 | 0 |
| **BDNF** | 0 | 0 | 0 |
| **EPO** | 0 | 0 | 0 |
| **PDGF-BB** | 0 | 0 | 0 |
| **VEGF-A** | 0 | 0 | 0 |

**Supplement Table 2S** Table shows all measured plasma factors at the three time points for newborns with severe ROP and with no ROP. Values are not corrected for gestational age.

|  |  | **ROP3** |  |  |  |  |  |  |  | **ROP0** |  |  |  |  |  |  |  |
| --- | --- | --- | --- | --- | --- | --- | --- | --- | --- | --- | --- | --- | --- | --- | --- | --- | --- |
| **cord blood** | **p** | **mean log10** | **sd log10** | **n** | **geometrical mean** | **lin mean** | **lin median** | **lin min** | **lin max** | **mean log10** | **sd log10** | **n** | **geometrical mean** | **lin mean** | **lin median** | **lin min** | **lin max** |
| **IL-1β** | 0.006 | 0.10 | 0.57 | 8 | 1.27 | 3.00 | 0.90 | 0.36 | 14.80 | -0.48 | 0.72 | 11 | 0.33 | 3.35 | 0.28 | 0.06 | 34.39 |
| **IL-2** | 0.890 | -0.32 | 0.59 | 8 | 0.47 | 1.23 | 0.19 | 0.19 | 6.27 | -0.34 | 0.46 | 11 | 0.45 | 0.76 | 0.19 | 0.19 | 2.43 |
| **IL-5** | 0.428 | 0.80 | 0.37 | 8 | 6.36 | 8.56 | 6.41 | 1.25 | 26.34 | 0.54 | 0.59 | 11 | 3.50 | 6.41 | 5.17 | 0.51 | 14.84 |
| **IL-6** | 0.407 | 0.58 | 0.87 | 8 | 3.80 | 25.96 | 1.66 | 0.56 | 171.07 | 0.23 | 0.62 | 11 | 1.70 | 8.44 | 1.03 | 0.56 | 78.33 |
| **IL-8** | 0.009 | 2.19 | 0.48 | 8 | 155.51 | 297.69 | 103.97 | 54.31 | 1341.78 | 1.74 | 0.49 | 11 | 55.26 | 151.59 | 46.50 | 13.19 | 1213.32 |
| **IL-10** | 0.086 | 0.51 | 0.71 | 8 | 3.20 | 9.50 | 4.90 | 0.21 | 52.06 | -0.14 | 0.78 | 11 | 0.73 | 3.24 | 0.21 | 0.21 | 18.09 |
| **IL-17** | 0.364 | 0.00 | 0.72 | 8 | 1.00 | 5.20 | 0.69 | 0.31 | 35.60 | -0.27 | 0.43 | 11 | 0.53 | 1.01 | 0.31 | 0.31 | 5.69 |
| **G-CSF** | 0.303 | 1.38 | 1.18 | 8 | 24.13 | 246.74 | 14.47 | 1.14 | 1334.18 | 0.91 | 1.11 | 11 | 8.16 | 84.96 | 1.14 | 1.14 | 442.30 |
| **IFN-γ** | 0.177 | 1.11 | 0.28 | 8 | 12.82 | 15.68 | 10.67 | 6.40 | 43.48 | 0.87 | 0.40 | 11 | 7.34 | 10.40 | 7.96 | 1.63 | 30.57 |
| **MCP-1** | 0.238 | 2.42 | 0.21 | 8 | 263.77 | 295.37 | 249.11 | 134.62 | 629.34 | 2.06 | 0.52 | 11 | 114.91 | 182.12 | 191.05 | 18.44 | 415.69 |
| **MIP-1β** | 0.041 | 1.99 | 0.30 | 8 | 96.97 | 117.90 | 89.28 | 32.27 | 250.23 | 1.65 | 0.29 | 11 | 44.92 | 53.53 | 54.53 | 11.45 | 106.63 |
| **TNF-α** | 0.125 | 1.78 | 0.53 | 8 | 60.44 | 107.65 | 62.66 | 6.61 | 391.37 | 1.62 | 0.15 | 11 | 41.75 | 43.66 | 44.50 | 19.44 | 67.11 |
| **IGF-1** | 0.043 | 1.08 | 0.34 | 8 | 12.10 | 16.00 | 9.50 | 5.00 | 38.00 | 1.43 | 0.16 | 11 | 26.92 | 28.73 | 26.00 | 15.00 | 50.00 |
| **BDNF** | 0.904 | 3.58 | 0.40 | 8 | 3784.22 | 5141.94 | 4285.84 | 608.47 | 12395.66 | 3.45 | 0.45 | 11 | 2811.83 | 3877.96 | 4137.35 | 281.98 | 7394.50 |
| **EPO** | 0.075 | 1.29 | 0.46 | 8 | 19.38 | 28.20 | 24.66 | 3.09 | 60.88 | 0.87 | 0.44 | 11 | 7.35 | 14.27 | 7.03 | 2.88 | 88.81 |
| **PDGF-BB** | 0.717 | 2.78 | 0.33 | 8 | 608.99 | 805.37 | 620.53 | 173.05 | 2674.06 | 2.72 | 0.36 | 11 | 520.25 | 648.04 | 718.32 | 72.89 | 1142.17 |
| **VEGF-A** | 0.657 | 2.49 | 0.58 | 8 | 307.48 | 569.25 | 470.02 | 61.36 | 1745.23 | 2.44 | 0.37 | 11 | 272.31 | 368.15 | 304.30 | 47.44 | 1110.75 |
|  |  |  |  |  |  |  |  |  |  |  |  |  |  |  |  |  |  |
| **day one** | **p** | **mean log10** | **sd log10** | **n** | **geometrical mean** | **lin mean** | **lin median** | **lin min** | **lin max** | **mean log10** | **sd log10** | **n** | **geometrical mean** | **lin mean** | **lin median** | **lin min** | **lin max** |
| **IL-1β** | 0.516 | -0.65 | 0.78 | 30 | 0.22 | 0.77 | 0.25 | 0.03 | 4.72 | -0.83 | 0.81 | 15 | 0.15 | 0.60 | 0.12 | 0.03 | 2.51 |
| **IL-2** | 0.508 | -0.15 | 0.62 | 30 | 0.71 | 1.89 | 0.41 | 0.19 | 9.55 | -0.07 | 0.59 | 15 | 0.85 | 1.89 | 0.67 | 0.19 | 7.07 |
| **IL-5** | 0.942 | 0.57 | 0.71 | 30 | 3.74 | 12.13 | 3.93 | 0.51 | 83.15 | 0.62 | 0.79 | 15 | 4.13 | 13.39 | 3.92 | 0.51 | 64.19 |
| **IL-6** | 0.942 | 0.95 | 1.14 | 30 | 8.88 | 155.09 | 8.07 | 0.09 | 2963.40 | 0.98 | 0.90 | 15 | 9.44 | 85.90 | 6.35 | 0.39 | 816.74 |
| **IL-8** | 0.528 | 2.06 | 0.68 | 30 | 113.75 | 1222.04 | 74.28 | 11.28 | 31241.26 | 1.87 | 0.43 | 15 | 74.23 | 128.99 | 64.67 | 20.19 | 705.28 |
| **IL-10** | 0.251 | 0.87 | 0.91 | 30 | 7.44 | 43.23 | 7.98 | 0.21 | 470.48 | 0.48 | 0.90 | 15 | 3.01 | 10.56 | 4.47 | 0.21 | 36.64 |
| **IL-17** | 0.687 | 0.14 | 0.66 | 30 | 1.39 | 3.90 | 1.38 | 0.31 | 19.64 | 0.05 | 0.62 | 15 | 1.13 | 2.80 | 0.62 | 0.31 | 12.09 |
| **G-CSF** | 1.000 | 1.89 | 1.40 | 30 | 77.10 | 4323.96 | 83.87 | 1.14 | 106037.17 | 1.93 | 1.05 | 15 | 84.38 | 573.84 | 91.86 | 1.14 | 3632.61 |
| **IFN-γ** | 0.310 | 0.81 | 0.69 | 30 | 6.43 | 25.40 | 5.50 | 0.41 | 171.38 | 1.01 | 0.65 | 15 | 10.28 | 30.13 | 9.45 | 1.24 | 129.56 |
| **MCP-1** | 0.482 | 2.19 | 0.72 | 30 | 154.40 | 666.07 | 117.82 | 10.91 | 6622.37 | 2.30 | 0.69 | 15 | 198.90 | 528.04 | 186.00 | 12.10 | 2086.35 |
| **MIP-1β** | 0.840 | 1.61 | 0.31 | 30 | 41.09 | 52.65 | 42.89 | 8.25 | 205.94 | 1.64 | 0.26 | 15 | 43.88 | 52.41 | 38.46 | 15.76 | 137.12 |
| **TNF-α** | 0.424 | 1.70 | 0.27 | 30 | 50.37 | 61.60 | 48.59 | 15.85 | 216.19 | 1.63 | 0.21 | 15 | 42.26 | 47.41 | 41.64 | 17.94 | 123.58 |
| **IGF-1** | 0.015 | 0.99 | 0.24 | 30 | 9.75 | 11.10 | 10.00 | 2.00 | 24.00 | 1.18 | 0.25 | 15 | 15.14 | 17.33 | 16.00 | 4.00 | 35.00 |
| **BDNF** | 0.006 | 3.26 | 0.42 | 30 | 1826.97 | 2697.80 | 1736.03 | 275.80 | 7468.99 | 3.62 | 0.30 | 15 | 4205.53 | 5091.54 | 4062.42 | 842.77 | 10454.55 |
| **EPO** | 0.576 | 1.03 | 0.47 | 30 | 10.68 | 24.37 | 8.83 | 2.34 | 231.13 | 1.07 | 0.45 | 15 | 11.82 | 19.14 | 11.62 | 1.89 | 69.40 |
| **PDGF-BB** | 0.058 | 2.63 | 0.37 | 30 | 425.66 | 585.24 | 471.55 | 65.95 | 2053.35 | 2.85 | 0.18 | 15 | 701.64 | 757.83 | 661.51 | 272.73 | 1230.24 |
| **VEGF-A** | 0.803 | 2.55 | 0.42 | 30 | 353.67 | 550.86 | 460.52 | 65.71 | 2426.28 | 2.53 | 0.42 | 15 | 338.92 | 500.28 | 408.17 | 71.00 | 1656.79 |
|  |  |  |  |  |  |  |  |  |  |  |  |  |  |  |  |  |  |
| **first month** | **p** | **mean log10** | **sd log10** | **n** | **geometrical mean** | **lin mean** | **lin median** | **lin min** | **lin max** | **mean log10** | **sd log10** | **n** | **geometrical mean** | **lin mean** | **lin median** | **lin min** | **lin max** |
| **IL-1β** | 0.954 | -0.50 | 0.88 | 30 | 0.31 | 2.64 | 0.38 | 0.03 | 38.64 | -0.57 | 0.67 | 16 | 0.27 | 0.69 | 0.39 | 0.03 | 4.30 |
| **IL-2** | 0.057 | -0.36 | 0.52 | 30 | 0.44 | 1.50 | 0.25 | 0.19 | 26.01 | -0.09 | 0.57 | 16 | 0.82 | 1.86 | 0.64 | 0.19 | 10.95 |
| **IL-5** | 0.720 | 0.33 | 0.46 | 30 | 2.15 | 5.28 | 2.13 | 0.51 | 84.36 | 0.43 | 0.58 | 16 | 2.71 | 6.75 | 2.32 | 0.51 | 37.53 |
| **IL-6** | 0.640 | 0.37 | 0.67 | 30 | 2.37 | 86.40 | 1.58 | 0.60 | 2515.33 | 0.24 | 0.73 | 16 | 1.75 | 14.44 | 1.54 | 0.09 | 198.94 |
| **IL-8** | 0.530 | 1.71 | 0.50 | 30 | 50.91 | 187.98 | 42.78 | 15.22 | 3810.27 | 1.61 | 0.39 | 16 | 40.51 | 68.27 | 31.66 | 15.78 | 401.32 |
| **IL-10** | 0.159 | 0.53 | 0.67 | 30 | 3.40 | 25.42 | 3.32 | 0.21 | 576.85 | 0.29 | 0.54 | 16 | 1.97 | 5.45 | 1.99 | 0.21 | 55.14 |
| **IL-17** | 0.452 | 0.22 | 0.59 | 30 | 1.65 | 4.55 | 1.50 | 0.31 | 56.89 | 0.34 | 0.48 | 16 | 2.21 | 3.74 | 2.47 | 0.31 | 18.40 |
| **G-CSF** | 0.466 | 0.76 | 0.88 | 30 | 5.82 | 137.22 | 2.64 | 1.14 | 3521.07 | 0.83 | 0.66 | 16 | 6.77 | 17.09 | 7.98 | 1.14 | 69.97 |
| **IFN-γ** | 0.500 | 0.89 | 0.32 | 30 | 7.82 | 11.87 | 8.25 | 2.80 | 116.99 | 0.95 | 0.37 | 16 | 8.95 | 14.04 | 6.91 | 2.28 | 84.08 |
| **MCP-1** | 0.973 | 2.15 | 0.34 | 30 | 141.23 | 260.79 | 126.40 | 48.13 | 3673.79 | 2.19 | 0.42 | 16 | 154.03 | 286.60 | 129.43 | 47.82 | 2114.01 |
| **MIP-1β** | 0.078 | 1.63 | 0.29 | 30 | 42.73 | 57.87 | 37.36 | 14.64 | 386.73 | 1.78 | 0.31 | 16 | 59.99 | 78.96 | 54.57 | 18.20 | 282.53 |
| **TNF-α** | 0.864 | 1.68 | 0.28 | 30 | 48.35 | 68.76 | 46.05 | 18.74 | 673.74 | 1.71 | 0.25 | 16 | 51.03 | 61.08 | 43.79 | 25.92 | 171.75 |
| **IGF-1** | 0.000 | 1.29 | 0.10 | 30 | 19.40 | 19.90 | 19.93 | 10.83 | 28.33 | 1.43 | 0.18 | 16 | 27.21 | 29.18 | 30.58 | 9.97 | 44.40 |
| **BDNF** | 0.126 | 3.69 | 0.34 | 30 | 4915.95 | 6281.50 | 5293.64 | 840.15 | 14951.79 | 3.83 | 0.36 | 16 | 6790.51 | 8593.47 | 7527.15 | 870.94 | 16669.66 |
| **EPO** | 0.165 | 0.93 | 0.22 | 30 | 8.54 | 9.81 | 7.90 | 2.73 | 39.94 | 0.99 | 0.16 | 16 | 9.67 | 10.33 | 9.48 | 4.97 | 21.63 |
| **PDGF-BB** | 0.331 | 2.95 | 0.34 | 30 | 885.43 | 1145.02 | 950.49 | 129.38 | 3622.09 | 3.04 | 0.21 | 16 | 1095.16 | 1199.36 | 1199.04 | 352.14 | 1948.13 |
| **VEGF-A** | 0.120 | 2.80 | 0.28 | 30 | 626.71 | 742.64 | 741.07 | 190.86 | 1392.22 | 2.95 | 0.24 | 16 | 881.68 | 1007.12 | 859.89 | 300.51 | 1921.49 |
|  |  |  |  |  |  |  |  |  |  |  |  |  |  |  |  |  |  |

**Supplement Table 3S BPD**. The table shows all measured serum factors at the three time points for newborns with and without BPD. Values are not corrected for gestational age.

|  |  | **with BPD** |  |  |  |  |  |  |  | **without BPD** | |  |  |  |  |  |  |
| --- | --- | --- | --- | --- | --- | --- | --- | --- | --- | --- | --- | --- | --- | --- | --- | --- | --- |
| **cord blood** | **p** | **mean log10** | **sd log10** | **n** | **geometrical mean** | **lin mean** | **lin median** | **lin min** | **lin max** | **mean log10** | **sd log10** | **n** | **geometrical mean** | **lin mean** | **lin median** | **lin min** | **lin max** |
| **IL-1β** | 0.295 | -0.10 | 0.64 | 12 | 0.80 | 2.25 | 0.62 | 0.06 | 14.80 | -0.32 | 0.58 | 16 | 0.48 | 2.60 | 0.36 | 0.13 | 34.39 |
| **IL-2** | 0.011 | -0.04 | 0.54 | 12 | 0.92 | 1.62 | 1.29 | 0.19 | 6.27 | -0.45 | 0.38 | 16 | 0.35 | 0.53 | 0.19 | 0.19 | 1.86 |
| **IL-5** | 0.068 | 0.91 | 0.38 | 12 | 8.16 | 10.73 | 9.48 | 1.25 | 26.34 | 0.60 | 0.51 | 16 | 3.94 | 6.11 | 5.17 | 0.51 | 14.84 |
| **IL-6** | 0.130 | 0.65 | 0.69 | 12 | 4.49 | 19.12 | 4.92 | 0.56 | 171.07 | 0.35 | 0.61 | 16 | 2.24 | 8.06 | 1.58 | 0.56 | 78.33 |
| **IL-8** | 0.272 | 2.10 | 0.50 | 12 | 124.69 | 239.20 | 108.61 | 13.19 | 1341.78 | 1.92 | 0.49 | 16 | 82.32 | 177.54 | 51.44 | 22.04 | 1213.32 |
| **IL-10** | 0.008 | 0.56 | 0.91 | 12 | 3.63 | 14.64 | 5.84 | 0.21 | 69.57 | 0.05 | 0.71 | 16 | 1.11 | 3.23 | 1.83 | 0.21 | 18.09 |
| **IL-17** | 0.149 | 0.09 | 0.68 | 12 | 1.23 | 4.68 | 1.07 | 0.31 | 35.60 | -0.22 | 0.42 | 16 | 0.60 | 1.03 | 0.31 | 0.31 | 5.69 |
| **G-CSF** | 0.072 | 1.46 | 1.09 | 12 | 29.12 | 201.71 | 57.61 | 1.14 | 1334.18 | 0.86 | 1.05 | 16 | 7.30 | 72.86 | 1.14 | 1.14 | 357.63 |
| **IFN-γ** | 0.304 | 1.24 | 0.41 | 12 | 17.35 | 25.23 | 20.58 | 4.64 | 63.99 | 1.00 | 0.35 | 16 | 9.92 | 12.85 | 9.70 | 2.04 | 30.57 |
| **MCP-1** | 0.245 | 2.48 | 0.50 | 12 | 301.14 | 470.23 | 395.14 | 18.44 | 1603.91 | 2.29 | 0.38 | 16 | 196.13 | 261.27 | 220.27 | 21.70 | 700.49 |
| **MIP-1β** | 0.168 | 1.98 | 0.43 | 12 | 94.92 | 135.45 | 97.57 | 11.45 | 314.66 | 1.75 | 0.27 | 16 | 56.83 | 67.87 | 59.71 | 19.56 | 189.50 |
| **TNF-α** | 0.264 | 1.86 | 0.39 | 12 | 71.62 | 104.54 | 65.86 | 19.44 | 391.37 | 1.65 | 0.24 | 16 | 44.62 | 49.32 | 47.70 | 6.61 | 79.37 |
| **IGF-1** | 0.272 | 1.23 | 0.22 | 12 | 17.02 | 19.08 | 18.00 | 8.00 | 40.00 | 1.29 | 0.34 | 16 | 19.51 | 24.63 | 23.00 | 5.00 | 50.00 |
| **BDNF** | 0.683 | 3.57 | 0.41 | 12 | 3692.74 | 5148.94 | 3988.26 | 580.25 | 13813.87 | 3.47 | 0.42 | 16 | 2959.24 | 4084.57 | 3917.14 | 281.98 | 12395.66 |
| **EPO** | 0.929 | 0.95 | 0.48 | 12 | 8.84 | 15.48 | 6.51 | 1.59 | 60.88 | 1.21 | 0.66 | 16 | 16.27 | 78.83 | 13.89 | 3.08 | 931.54 |
| **PDGF-BB** | 0.958 | 2.87 | 0.31 | 12 | 737.07 | 900.60 | 719.94 | 152.07 | 1816.68 | 2.74 | 0.36 | 16 | 543.26 | 719.69 | 608.31 | 72.89 | 2674.06 |
| **VEGF-A** | 0.204 | 2.50 | 0.57 | 12 | 315.19 | 597.39 | 359.85 | 19.45 | 2219.55 | 2.43 | 0.49 | 16 | 271.06 | 460.40 | 300.82 | 47.44 | 1745.23 |
|  |  |  |  |  |  |  |  |  |  |  |  |  |  |  |  |  |  |
| **day one** | **p** | **mean log10** | **sd log10** | **n** | **geometrical mean** | **lin mean** | **lin median** | **lin min** | **lin max** | **mean log10** | **sd log10** | **n** | **geometrical mean** | **lin mean** | **lin median** | **lin min** | **lin max** |
| **IL-1β** | 0.903 | -0.75 | 0.73 | 33 | 0.18 | 0.56 | 0.19 | 0.03 | 2.57 | -0.82 | 0.81 | 35 | 0.15 | 0.63 | 0.10 | 0.03 | 4.49 |
| **IL-2** | 0.786 | -0.15 | 0.59 | 33 | 0.71 | 1.71 | 0.38 | 0.19 | 8.10 | -0.17 | 0.60 | 35 | 0.68 | 1.72 | 0.34 | 0.19 | 9.55 |
| **IL-5** | 0.764 | 0.62 | 0.66 | 33 | 4.20 | 11.42 | 4.07 | 0.51 | 83.15 | 0.52 | 0.75 | 35 | 3.27 | 11.65 | 3.46 | 0.51 | 64.19 |
| **IL-6** | 0.871 | 0.99 | 0.97 | 33 | 9.87 | 137.97 | 6.37 | 0.09 | 2963.40 | 0.92 | 1.00 | 35 | 8.37 | 109.32 | 6.08 | 0.09 | 1884.34 |
| **IL-8** | 0.309 | 2.04 | 0.57 | 33 | 109.22 | 1080.50 | 72.05 | 21.76 | 31241.26 | 1.89 | 0.56 | 35 | 77.90 | 195.15 | 64.67 | 11.28 | 1359.94 |
| **IL-10** | 0.828 | 0.73 | 0.88 | 33 | 5.38 | 25.97 | 7.20 | 0.21 | 306.05 | 0.55 | 0.89 | 35 | 3.54 | 23.84 | 4.45 | 0.21 | 470.48 |
| **IL-17** | 0.640 | 0.04 | 0.62 | 33 | 1.09 | 3.02 | 0.62 | 0.31 | 16.40 | 0.01 | 0.62 | 35 | 1.01 | 2.83 | 0.31 | 0.31 | 19.64 |
| **G-CSF** | 0.428 | 1.86 | 1.33 | 33 | 72.75 | 3906.87 | 100.62 | 1.14 | 106037.17 | 1.56 | 1.30 | 35 | 36.61 | 506.82 | 52.09 | 1.14 | 3632.61 |
| **IFN-γ** | 0.604 | 0.89 | 0.60 | 33 | 7.69 | 20.38 | 6.80 | 0.14 | 171.38 | 0.82 | 0.76 | 35 | 6.56 | 26.41 | 6.26 | 0.15 | 161.60 |
| **MCP-1** | 0.655 | 2.25 | 0.56 | 33 | 177.84 | 411.20 | 142.48 | 14.21 | 3328.75 | 2.19 | 0.75 | 35 | 154.50 | 622.67 | 168.40 | 10.91 | 6622.37 |
| **MIP-1β** | 0.897 | 1.60 | 0.26 | 33 | 39.90 | 47.88 | 43.25 | 14.22 | 148.12 | 1.57 | 0.32 | 35 | 37.44 | 48.81 | 38.06 | 8.25 | 205.94 |
| **TNF-α** | 0.600 | 1.68 | 0.26 | 33 | 47.82 | 57.88 | 50.18 | 17.94 | 216.19 | 1.63 | 0.21 | 35 | 42.33 | 47.68 | 39.81 | 15.85 | 141.27 |
| **IGF-1** | 0.184 | 1.04 | 0.20 | 33 | 10.91 | 12.24 | 10.00 | 5.00 | 38.00 | 1.07 | 0.30 | 35 | 11.73 | 14.49 | 12.00 | 2.00 | 47.00 |
| **BDNF** | 0.022 | 3.27 | 0.48 | 33 | 1856.75 | 2938.56 | 2346.43 | 117.08 | 7612.80 | 3.43 | 0.38 | 35 | 2710.07 | 3715.81 | 2975.92 | 455.82 | 10454.55 |
| **EPO** | 0.580 | 0.98 | 0.42 | 33 | 9.52 | 16.82 | 8.91 | 1.93 | 166.66 | 1.05 | 0.54 | 35 | 11.31 | 28.26 | 8.12 | 1.89 | 231.13 |
| **PDGF-BB** | 0.153 | 2.59 | 0.45 | 33 | 393.42 | 576.89 | 581.86 | 28.35 | 2053.35 | 2.72 | 0.28 | 35 | 527.73 | 625.59 | 553.08 | 66.00 | 1375.18 |
| **VEGF-A** | 0.494 | 2.53 | 0.43 | 33 | 341.50 | 528.34 | 418.89 | 65.71 | 2198.68 | 2.56 | 0.38 | 35 | 360.67 | 521.97 | 434.35 | 71.00 | 2426.28 |
|  |  |  |  |  |  |  |  |  |  |  |  |  |  |  |  |  |  |
| **first month** | **p** | **mean log10** | **sd log10** | **n** | **geometrical mean** | **lin mean** | **lin median** | **lin min** | **lin max** | **mean log10** | **sd log10** | **n** | **geometrical mean** | **lin mean** | **lin median** | **lin min** | **lin max** |
| **IL-1β** | 0.897 | -0.46 | 0.84 | 32 | 0.34 | 2.53 | 0.38 | 0.03 | 38.64 | -0.78 | 0.64 | 36 | 0.17 | 0.52 | 0.12 | 0.03 | 4.37 |
| **IL-2** | 0.222 | -0.30 | 0.56 | 32 | 0.50 | 1.70 | 0.27 | 0.19 | 26.01 | -0.27 | 0.42 | 36 | 0.53 | 0.98 | 0.53 | 0.19 | 10.95 |
| **IL-5** | 0.721 | 0.35 | 0.52 | 32 | 2.22 | 6.05 | 1.69 | 0.51 | 84.36 | 0.40 | 0.41 | 36 | 2.52 | 4.13 | 2.40 | 0.51 | 37.53 |
| **IL-6** | 0.906 | 0.36 | 0.65 | 32 | 2.28 | 81.02 | 1.69 | 0.35 | 2515.33 | 0.25 | 0.55 | 36 | 1.78 | 7.97 | 1.42 | 0.09 | 198.94 |
| **IL-8** | 0.897 | 1.70 | 0.46 | 32 | 50.30 | 175.99 | 43.38 | 16.95 | 3810.27 | 1.59 | 0.35 | 36 | 39.09 | 60.07 | 35.44 | 15.22 | 401.32 |
| **IL-10** | 0.022 | 0.62 | 0.65 | 32 | 4.15 | 25.13 | 3.69 | 0.21 | 576.85 | 0.28 | 0.54 | 36 | 1.92 | 4.63 | 2.06 | 0.21 | 55.14 |
| **IL-17** | 0.828 | 0.22 | 0.61 | 32 | 1.66 | 4.58 | 1.56 | 0.31 | 56.89 | 0.21 | 0.46 | 36 | 1.61 | 2.84 | 1.41 | 0.31 | 18.40 |
| **G-CSF** | 0.755 | 0.81 | 0.81 | 32 | 6.43 | 127.59 | 4.63 | 1.14 | 3521.07 | 0.69 | 0.70 | 36 | 4.86 | 16.32 | 2.97 | 1.14 | 90.78 |
| **IFN-γ** | 0.838 | 0.88 | 0.33 | 32 | 7.57 | 11.65 | 6.05 | 1.90 | 116.99 | 0.90 | 0.32 | 36 | 7.86 | 11.12 | 7.44 | 2.28 | 84.08 |
| **MCP-1** | 0.715 | 2.13 | 0.35 | 32 | 133.88 | 248.34 | 123.18 | 46.21 | 3673.79 | 2.16 | 0.31 | 36 | 143.86 | 211.80 | 133.82 | 48.13 | 2114.01 |
| **MIP-1β** | 0.519 | 1.64 | 0.29 | 32 | 43.78 | 58.60 | 40.57 | 14.64 | 386.73 | 1.66 | 0.28 | 36 | 45.65 | 57.88 | 46.50 | 18.20 | 282.53 |
| **TNF-α** | 0.699 | 1.68 | 0.27 | 32 | 47.62 | 67.15 | 44.69 | 21.63 | 673.74 | 1.64 | 0.18 | 36 | 43.64 | 48.05 | 43.30 | 18.74 | 171.75 |
| **IGF-1** | 0.389 | 1.32 | 0.13 | 32 | 20.78 | 21.65 | 20.56 | 10.83 | 36.79 | 1.37 | 0.14 | 36 | 23.59 | 24.86 | 23.38 | 9.97 | 44.40 |
| **BDNF** | 0.064 | 3.70 | 0.34 | 32 | 5046.67 | 6456.67 | 5322.20 | 840.15 | 16294.97 | 3.82 | 0.33 | 36 | 6566.77 | 8230.29 | 7232.50 | 824.02 | 20738.31 |
| **EPO** | 0.366 | 0.96 | 0.23 | 32 | 9.04 | 10.56 | 8.73 | 3.98 | 39.94 | 0.95 | 0.22 | 36 | 8.82 | 10.05 | 7.90 | 2.73 | 27.46 |
| **PDGF-BB** | 0.120 | 2.95 | 0.29 | 32 | 898.52 | 1082.54 | 991.23 | 129.38 | 3062.04 | 3.01 | 0.30 | 36 | 1017.13 | 1222.31 | 1344.25 | 102.71 | 3622.09 |
| **VEGF-A** | 0.629 | 2.83 | 0.24 | 32 | 671.93 | 760.26 | 730.07 | 190.86 | 1612.21 | 2.86 | 0.29 | 36 | 731.41 | 879.08 | 859.89 | 154.61 | 1921.49 |

**Methods – details on correlation analysis**

Calculations of Spearman’s rank correlation coefficients was based on the cor function in R with method spearman. using the function corrplot.mixed in the R package corrplot version 0.84 for graphical presentation; alternatively. the function rcorr (with type spearman) in package Hmisc version 4.0.4 was used to determine correlation coefficients and their probability levels. Comparison of correlations and calculation of z values was performed using the function paired.r in package psych version 2.1.3; this function performs a test on z-transformed values of r. Again, the function corrplot.mixed was used to present the result. Heatmaps on sum of z-values of differences in correlations were generated using the package ComplexHeatmap version 2.6.2. Outcome-related networks (networks based on between-outcome group correlation differences) were constructed from correlations significantly different between the groups with and without outcome; these networks were presented as heatmaps for z-scores and network images. Total networks included all within-outcome significant correlations and were presented only as heatmaps for z-scores. In all cases. P < 0.05 was considered significant.

As GA at birth is the single confounding factor with most pronounced effect on neonatal morbidities. statistical correction was made by partial correlation analysis. For every cytokine from all full sets of data after interpolation. a linear model was fitted between log cytokine concentration and GA. The normalized residuals were subsequently used for analysis of levels of individual cytokines as well as of correlations between cytokines for the various groups.
